# Driving Among High School Students — United States, 2013

**Published:** 2015-04-03

**Authors:** Ruth A. Shults, Emily Olsen, Allan F. Williams

**Affiliations:** 1 Division of Unintentional Injury Prevention, National Center for Injury Prevention and Control, CDC; 2 Division of Adolescent and School Health, National Center for HIV/AIDS, Viral Hepatitis, STD, and TB Prevention, CDC; 3 Allan F. Williams, Bethesda, Maryland

During 2004–2013, the number of passenger vehicle drivers aged 16–19 years involved in fatal crashes in the United States declined by 55% from 5,724 to 2,568.* In addition to graduated driver licensing (GDL) programs (1) and safer vehicles,† other possible contributors to the decline include adolescents waiting longer to get their driver licenses and driving less (2). The crash risk for drivers of any age is highest during the first months of independent driving, and this risk is highest for the youngest teenage drivers (3). To estimate the percentage of high school students aged ≥16 years who have driven during the past 30 days, by age, race/ethnicity, and location, CDC analyzed 2013 data from the national Youth Risk Behavior Survey (YRBS) and YRBS data collected by 42 states and 21 large urban school districts. Nationwide, 76.3% of high school students aged ≥16 years reported having driven during the 30 days before the survey; 83.2% of white students had driven compared with <70% of black and Hispanic students. Across 42 states, the percentage of students who drove ranged from 53.8% to 90.2%. Driving prevalence was higher in the midwestern and mountain states. Across the 21 large urban school districts, the percentage of drivers varied more than twofold from 30.2% to 76.0%. This report provides the most detailed evidence to date that the percentage of students who drive varies substantially depending on where they live. Such information will be vital as states and communities consider potential ways to improve safety for older teenage novice drivers and plan for safe, affordable transportation options for those who do not drive.

The 2013 national YRBS used a three-stage cluster sample to obtain cross-sectional data representative of public and private school students in grades 9–12 in all 50 states and the District of Columbia (4). The usable sample size was 13,583, with a 68% overall response rate.§ The state and large urban school district YRBSs used two-stage cluster samples to obtain cross-sectional data representative of public school students in grades 9–12 in 39 states and 21 districts and of public and private school students in grades 9–12 in three states (Ohio, South Dakota, and Vermont). Sample sizes across states ranged from 1,107 to 53,785, and overall response rates ranged from 60% to 87%. Sample sizes across large urban school districts ranged from 1,102 to 10,778, and overall response rates ranged from 69% to 90%. Data by race/ethnicity are presented for non-Hispanic black, non-Hispanic white, and Hispanic students.

Respondents completed a voluntary, anonymous, self-administered questionnaire that included questions about drinking and driving and questions about texting and driving. In 2013, for the first time, these questions included a response option of “I did not drive a car or other vehicle during the past 30 days.” For this report, driving was defined as having responded to the question about drinking and driving or the question about texting and driving with a response other than “I did not drive a car or other vehicle during the past 30 days.” Data were weighted to provide estimates at the national, state, or large urban school district level, and statistical software was used to account for the complex sample designs. All analyses were conducted among students aged ≥16 years, the age at which persons in every jurisdiction except New Jersey and New York City, New York, could be licensed to drive independently.¶ Chi-square tests were used to test for significant (p<0.05) differences among subgroups for the national data.

Nationwide, 76.3% of U.S. high school students aged ≥16 years reported having driven during the 30 days before the survey (Table 1); 83.2% of white students had driven, compared with 67.6% of black students and 68.9% of Hispanic students. The percentage of students who had driven increased with age from 69.8% for students aged 16 years to 84.2% for those aged ≥18 years. Across the 42 state surveys, the percentage of drivers ranged from 53.8% in Hawaii to 90.2% in South Dakota (median: 80.8%) (Table 2). Among students aged ≥18 years, the percentage who had driven varied from 57.9% in Hawaii to 94.9% in North Dakota (median: 84.4%). Driving prevalence was higher in the midwestern and mountain states compared with other regions of the country (Figure). Across the 21 districts, the percentage of drivers ranged from 30.2% in San Francisco, California, to 76.0% in Charlotte-Mecklenburg, North Carolina (median: 57.7%) (Table 2).

## Discussion

This report indicates that, nationwide, three out of four U.S. high school students aged ≥16 years drove at least once during the 30 days before the survey, and the percentage who drove varied substantially depending on where they lived. The percentage of students who drove was higher in the midwestern and mountain states, where population density is relatively low** and alternative transportation options might be limited (5). The lower percentage of student drivers in metropolitan areas compared with states (median: 57.7% versus 80.8%) might be related to family income, shorter travel distances, and wider use of transportation alternatives including walking, bicycling, and taking public transportation (5–8). The finding that in some states and most metropolitan areas at least 20% of students aged ≥18 years did not drive has implications for how they will learn to drive. For example, most students are supervised during the learning period by a parent or guardian (9). If they do not learn to drive before they leave home, their opportunities for practice driving with a supervisor might be more limited.

The racial/ethnic disparities found in the percentage of teenage drivers are consistent with findings from previous research (2,6,7). For example, a 2010 survey of U.S. high school seniors reported that the percentage of black students who were unlicensed was twice the percentage of white students (39% versus 16%), and they were more than twice as likely to not drive in an average week as white students (37% versus 14%) (2). Reaching adulthood without having obtained a driver license might limit educational, housing, and employment options.

Declines in licenses and driving among teenagers have coincided with the economic recession of the mid-2000s and have not rebounded (2), raising concern that teenagers from lower income families might find that meeting the requirements for licensure is becoming increasingly difficult (6,7). Stated reasons for delaying licensure support this concern, including not having access to a car and the costs of driving (7,10). GDL programs are designed to provide teenagers with a protective learning environment through supervised practice driving and by restricting nighttime driving and the number and age of passengers allowed during the first months of independent driving. However, in nearly every state, GDL programs apply only to novice drivers aged <18 years. Therefore, persons who do not obtain a license before their 18th birthday, many of whom are from low income or minority families, do not participate in the GDL program. Research regarding the potential safety benefits and risks associated with teenagers getting licensed after their 18th birthday is being conducted. Some researchers have suggested that extending GDL requirements to novice drivers aged 18–20 years might provide safety benefits, particularly for low income and minority youths (1,6,7).

The findings in this report are subject to at least seven limitations. First, neither licensure status nor whether teens were driving independently or under adult supervision was assessed. Second, state- and district-level percentages of drivers stratified by race/ethnicity were not presented because of small numbers. Third, the data were self-reported, and the extent of any underreporting or overreporting cannot be determined. Fourth, data were not available for eight states, including the west coast states of Washington, Oregon, and California. Fifth, the participating large urban schools districts were clustered on the east and west coasts, resulting in limited representation from districts in the midwestern and mountain regions. Sixth, results are not representative of high school–aged youths who do not attend high school. Finally, the data were weighted to adjust for school and student nonresponse and the distribution of students by grade, sex, and race/ethnicity in each jurisdiction. Nonetheless, nonresponse bias is possible and might have affected the results.

What is already known on this topic?

Teenagers in the United States are waiting longer to get their driver licenses and driving less. Racial/ethnic and income disparities exist in teen licensure rates and driving experience. The potential safety benefits and risks associated with teenagers getting licensed after their 18th birthday are not well understood.

What is added by this report?

Data from the 2013 national Youth Risk Behavior Survey indicate that 76.3% of high school students nationwide aged ≥16 years drove during the 30 days before the survey; 83.2% of white students had driven compared with <70% of black and Hispanic students. Across 42 states, the percentage of drivers ranged from 53.8% in Hawaii to 90.2% in South Dakota. The prevalence of driving was higher in the midwestern and mountain states. Across 21 large urban school districts, the percentage of drivers varied from 30.2% in San Francisco, California, to 76.0% in Charlotte-Mecklenburg, North Carolina.

What are the implications for public health practice?

The number of persons who reach age 18 years with little or no driving experience is substantial, especially among blacks and Hispanics and in certain metropolitan areas. Because the age at which persons begin driving varies substantially by location, strategies to address transportation needs among teenagers could benefit from considering their local driving patterns. The data provide a baseline for future studies of driving trends among teenagers, which can aid states and communities in considering ways to improve safety for older novice teenage drivers and in planning for safe, affordable transportation options for teenagers who do not drive.

This report provides previously unavailable information on driving among U.S. adolescents by state and metropolitan area. The data reveal substantial variations in driving patterns across the country and provide a baseline for future studies measuring trends. As driving practices among adolescents continue to evolve, such information can aid states and communities in considering potential ways to improve safety for older teenage novice drivers. In addition, these results support the need for safe, affordable transportation options for teenagers who do not drive, especially for those who face economic barriers to licensing.

## Figures and Tables

**FIGURE:**
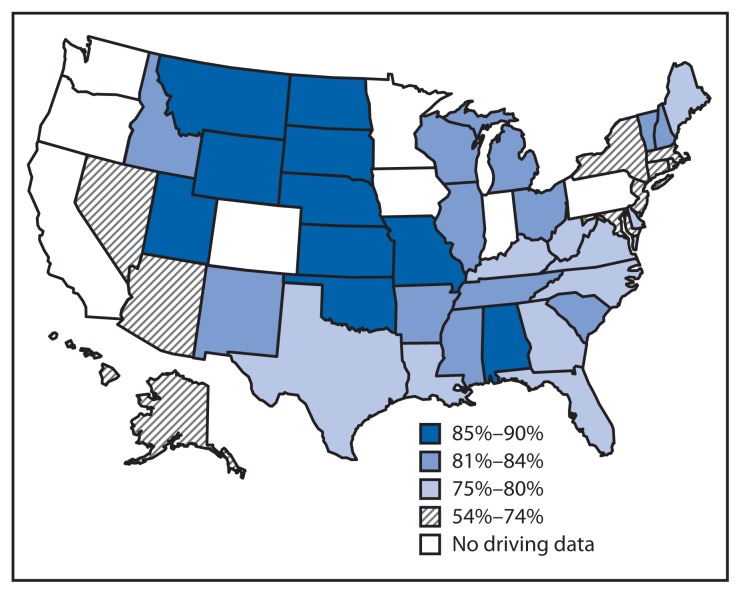
Percentage of high school students aged ≥16 years who reported driving a car or other vehicle during the 30 days before the survey — Youth Risk Behavior Surveys, 42 states,* 2013 * Data were not available for California, Colorado, Indiana, Iowa, Minnesota, Oregon, Pennsylvania, and Washington.

**TABLE 1:** Percentage of high school students aged ≥16 years who reported driving a car or other vehicle during the 30 days before the survey — national Youth Risk Behavior Survey, United States, 2013

Characteristic	%	95% CI
**Total**	**76.3**	**73.4–79.0**
**Sex** *		
Male	78.3	74.9–81.3
Female	74.2	71.3–76.9
Race/Ethnicity*,†		
White, non-Hispanic	83.2	80.7–85.4
Black, non-Hispanic	67.6	63.8–71.1
Hispanic	68.9	66.0–71.6
**Age (yrs)** *		
16	69.8	65.8–73.4
17	78.0	74.8–80.9
≥18	84.2	81.2–86.7

**Abbreviation:** CI = confidence interval.

* Chi-square test, p<0.05.

† The numbers of students from other racial/ethnic groups were too small for meaningful analysis.

**TABLE 2:** Percentage of high school students aged ≥16 years who reported driving a car or other vehicle during the 30 days before the survey, by age — Youth Risk Behavior Surveys, 42 states and 21 large urban school districts,* 2013

	≥16 yrs		16 yrs		17 yrs		≥18 yrs	
				
Site	%	95% CI	%	95% CI	%	95% CI	%	95% CI
**State surveys**								
Alabama	88.5	83.6–92.1	84.3	76.8–89.8	92.3	86.1–95.8	90.0	84.6–93.7
Alaska	73.0	69.2–76.5	63.2	57.0–68.9	77.5	72.7–81.6	81.3	71.2–88.4
Arizona	68.4	62.2–74.0	67.1	60.6–73.1	66.6	58.2–74.1	72.7	63.5–80.3
Arkansas	82.6	77.5–86.7	80.2	71.4–86.7	81.7	77.3–85.5	87.5	79.6–92.6
Connecticut	67.7	63.8–71.4	55.4	50.5–60.1	75.3	70.2–79.8	78.0	70.0–84.4
Delaware	80.2	77.5–82.6	74.6	70.3–78.4	85.6	82.6–88.2	82.6	76.4–87.4
Florida	74.8	72.6–76.8	69.2	66.4–71.9	75.8	72.9–78.4	82.7	79.5–85.5
Georgia	74.7	69.7–79.2	70.8	65.0–75.9	73.5	64.6–80.9	82.1	77.0–86.3
Hawaii	53.8	49.4–58.2	43.6	38.0–49.3	63.1	58.0–67.8	57.9	49.1–66.2
Idaho	84.0	81.4–86.3	82.0	78.7–84.9	85.0	80.7–88.6	85.7	80.2–89.9
Illinois	80.8	76.8–84.3	79.0	73.0–83.9	80.9	76.3–84.7	83.6	77.1–88.6
Kansas	86.1	83.2–88.6	81.1	77.3–84.4	88.4	83.3–92.0	91.6	85.7–95.2
Kentucky	77.7	72.4–82.2	73.1	66.4–78.9	79.8	73.2–85.0	82.7	71.5–90.1
Louisiana	78.7	74.6–82.3	75.4	66.9–82.3	80.7	75.8–84.8	80.8	69.7–88.5
Maine	75.8	74.0–77.4	71.9	69.7–74.0	79.8	77.4–82.1	75.7	72.9–78.3
Maryland	65.9	64.6–67.2	58.1	56.6–59.6	71.4	69.7–73.0	73.0	70.9–75.0
Massachusetts	66.1	61.9–70.0	53.3	48.6–58.0	73.2	66.9–78.8	78.2	72.5–83.0
Michigan	82.4	78.7–85.6	76.6	71.8–80.8	86.0	81.4–89.6	87.4	82.9–90.8
Mississippi	83.6	78.0–88.0	79.7	70.7–86.5	87.0	80.5–91.6	87.4	81.4–91.6
Missouri	84.7	79.1–89.0	83.4	77.4–88.0	83.8	76.8–89.0	88.7	73.5–95.7
Montana	88.7	87.2–90.2	85.5	83.0–87.7	89.4	87.0–91.4	93.2	90.6–95.1
Nebraska	87.5	84.0–90.3	85.4	80.0–89.6	89.2	84.3–92.7	—†	—
Nevada	71.1	66.2–75.6	61.4	55.4–67.2	74.8	67.4–80.9	82.6	77.3–86.9
New Hampshire	81.8	78.5–84.8	77.6	72.4–82.1	83.5	79.4–87.0	86.6	81.6–90.4
New Jersey	70.5	65.8–74.8	49.8	42.7–56.8	82.8	76.8–87.5	85.5	81.4–88.8
New Mexico	80.8	75.7–85.0	78.7	74.7–82.1	81.9	73.8–88.0	84.8	79.5–88.9
New York	62.4	56.1–68.2	53.2	46.4–59.9	64.7	57.1–71.6	77.5	68.2–84.7
North Carolina	77.6	71.1–82.9	72.1	64.5–78.5	79.7	70.5–86.5	83.8	76.9–89.0
North Dakota	89.7	87.3–91.7	84.1	79.2–88.0	91.7	89.0–93.9	94.9	91.6–97.0
Ohio	81.3	75.6–85.9	78.0	70.1–84.2	80.6	74.6–85.6	88.6	82.1–92.9
Oklahoma	85.1	82.2–87.5	78.1	72.0–83.2	87.4	83.7–90.4	92.8	85.7–96.5
Rhode Island	69.9	63.5–75.7	57.5	48.9–65.6	78.0	70.9–83.7	82.6	77.0–87.1
South Carolina	82.6	78.5–86.0	78.4	73.1–82.9	82.4	74.2–88.4	89.8	83.1–94.1
South Dakota	90.2	87.5–92.3	85.6	79.8–90.0	94.6	91.8–96.6	90.3	82.6–94.8
Tennessee	81.1	76.9–84.7	77.3	70.7–82.7	83.2	77.5–87.8	84.2	77.1–89.4
Texas	78.0	74.3–81.2	69.2	62.2–75.4	80.4	77.4–83.1	88.4	85.7–90.6
Utah	88.1	83.8–91.4	84.7	79.0–89.1	89.2	83.9–92.9	93.0	86.3–96.6
Vermont	82.6	80.6–84.5	79.6	76.9–82.0	84.9	82.0–87.3	84.5	81.7–86.9
Virginia	76.9	73.9–79.6	73.1	69.6–76.3	79.8	75.7–83.4	81.1	75.0–86.0
West Virginia	80.4	77.3–83.2	76.6	72.1–80.6	82.0	76.9–86.2	84.0	77.0–89.2
Wisconsin	83.4	79.9–86.4	77.5	72.5–81.9	86.3	81.5–90.1	87.7	81.8–91.9
Wyoming	86.9	84.2–89.3	85.1	80.9–88.5	87.9	84.7–90.5	88.6	83.6–92.3
**Large urban school district surveys**								
Baltimore, Maryland	54.5	49.5–59.3	46.9	39.2–54.8	60.6	55.1–65.9	59.0	48.7–68.7
Boston, Massachusetts	33.8	29.7–38.1	24.5	20.0–29.6	33.7	27.9–40.1	42.2	35.0–49.7
Broward County, Florida	73.1	69.1–76.8	65.9	59.5–71.7	76.6	71.0–81.3	80.2	72.7–86.0
Charlotte-Mecklenburg, North Carolina	76.0	72.4–79.3	64.3	58.0–70.1	81.9	76.5–86.3	84.9	78.8–89.5
Chicago, Illinois	49.1	46.1–52.2	37.7	33.2–42.4	53.5	47.0–59.9	60.5	54.2–66.6
Detroit, Michigan	65.5	60.3–70.2	58.5	49.8–66.8	70.0	63.8–75.5	71.0	63.1–77.8
District of Columbia	42.6	41.1–44.1	40.4	38.3–42.4	41.7	39.3–44.0	51.3	47.6–55.1
Duval County, Florida	75.1	72.7–77.3	71.2	67.9–74.3	76.4	72.8–79.6	79.9	72.6–85.6
Houston, Texas	70.5	67.0–73.7	67.7	62.0–72.8	68.8	63.3–73.8	76.4	71.8–80.4
Los Angeles, California	48.8	43.8–53.9	41.2	32.4–50.6	51.1	43.0–59.2	58.3	51.9–64.4
Memphis, Tennessee	67.8	64.0–71.4	56.5	51.0–61.8	74.1	67.9–79.5	77.9	70.7–83.7
Miami-Dade County, Florida	65.8	61.9–69.6	58.4	53.2–63.5	68.0	61.6–73.8	73.6	67.9–78.7
Milwaukee, Wisconsin	54.5	51.3–57.8	50.8	45.9–55.7	57.9	51.4–64.1	55.4	47.8–62.8
New York City, New York	31.0	28.2–33.9	27.0	22.3–32.3	33.3	30.3–36.4	39.8	33.6–46.2
Orange County, Florida	67.5	63.4–71.3	62.1	55.6–68.2	68.8	63.5–73.6	75.4	68.3–81.4
Palm Beach County, Florida	73.5	70.6–76.2	69.9	65.5–74.0	72.8	68.0–77.1	79.4	73.0–84.7
Philadelphia, Pennsylvania	47.7	43.1–52.3	45.1	39.7–50.7	46.8	40.3–53.5	53.0	42.1–63.6
San Bernardino, California	59.6	55.1–64.0	54.9	48.9–60.7	61.5	52.5–69.9	—†	—
San Diego, California	57.7	52.9–62.3	50.9	45.0–56.8	60.2	53.0–67.1	68.5	60.9–75.3
San Francisco, California	30.2	27.0–33.7	24.1	19.5–29.4	32.0	27.5–36.8	39.2	32.5–46.4
Seattle, Washington	54.0	49.8–58.1	51.1	45.0–57.2	55.8	49.7–61.8	57.8	47.0–67.9

**Abbreviation:** CI = confidence interval.

* Data were not available for California, Colorado, Indiana, Iowa, Minnesota, Oregon, Pennsylvania, and Washington. Data were collected from public school students in 39 states and 21 large urban school districts and from public and private school students in three states.

† Estimate suppressed because cell size was <100.

## Footnotes

* Available at http://www.iihs.org/iihs/topics/t/teenagers/fatalityfacts/teenagers.

† Available at http://www-nrd.nhtsa.dot.gov/Pubs/812020.pdf.

§ Overall response rate = (number of participating schools/number of eligible sampled schools) × (number of usable questionnaires/number of eligible students sampled).

¶ Available at http://www.iihs.org/iihs/topics/laws/graduatedlicenseintro?topicName=teenagers.

** Available at http://www2.census.gov/geo/pdfs/maps-data/maps/thematic/us_popdensity_2010map.pdf.
